# Healthcare and Epidemiological Surveillance Costs of Hepatitis A Outbreaks in Spain in Regions with and without Universal Hepatitis A Vaccination of Children during 2010-2018

**DOI:** 10.3390/vaccines12060648

**Published:** 2024-06-11

**Authors:** Pedro Plans-Rubió, Carles Pericas, Ana Maria Avellon, Concepción Izquierdo, Ana Martínez, Núria Torner, Alejandro Martínez, Eva Borrás, Francisco Roig, Pere Godoy, Cristina Rius

**Affiliations:** 1 Department of Health of Catalonia, Public Health Agency of Catalonia, 08005 Barcelona, Spain; 2 Ciber of Epidemiology and Public Health (CIBERESP), 28028 Madrid, Spain; 3 Institut de Recerca Biomèdica, Hospital Sant Pau, 08041 Barcelona, Spain; 4 Hepatitis Unit, Centro Nacional de Microbiología, Instituto de Salud Carlos III, 28222 Madrid, Spain; 5 Servicio de Epidemiología, Consejería de Salud de la Región de Murcia, 30008 Murcia, Spain; 6 Departamento de Medicina, Universidad de Barcelona, 08036 Barcelona, Spain; 7 Subdirección General de Epidemiología y Vigilancia de la Salud, Comunidad Valenciana, 46020 Valencia, Spain; roig_fco@gva.es; 8 Institut de Recerca Biomèdica (IRB Lleida), 25198 Lleida, Spain; 9 Agencia de Salud Pública de Barcelona, 08023 Barcelona, Spain

**Keywords:** hepatitis A vaccine, hepatitis A prevention, hepatitis A outbreak-associated costs, epidemiological surveillance costs, healthcare costs, hepatitis A outbreaks

## Abstract

The aim of this study was to evaluate and compare hepatitis A outbreak-associated healthcare and epidemiological surveillance costs in Spain in two types of autonomous regions during 2010–2018: (1) regions with a prevention strategy based on universal hepatitis A vaccination of children and vaccination of high-risk population groups (Catalonia) and (2) regions with a prevention strategy based on vaccinating high-risk population groups (Castile and Leon, Murcia, Navarra, Community of Madrid, Community of Valencia). Healthcare costs were determined based on the resources used to treat hepatitis A outbreak-associated cases and hospitalizations. Epidemiological surveillance costs were calculated from the resources used during surveillance activities. The ratios for total, healthcare and epidemiological surveillance costs (regions without universal hepatitis A vaccination of children vs. Catalonia) were used to compare the two hepatitis A prevention strategies. From 2010 to 2018, the total, healthcare and epidemiological surveillance costs per million population were 1.75 times (EUR 101,671 vs. EUR 58,032), 1.96 times (EUR 75,500 vs. EUR 38,516) and 1.34 times greater (EUR 26,171 vs. EUR 19,515) in regions without universal hepatitis A vaccination of children than in Catalonia, respectively. The ratios tended to increase over time during 2010–2018. In 2015–2018, total, healthcare and epidemiological surveillance costs per million population were 2.68 times (EUR 69,993 vs. EUR 26,158), 2.86 times (EUR 53,807 vs. EUR 18,825) and 2.21 times greater (EUR 16,186 vs. EUR 7333) in regions without universal hepatitis A vaccination of children than in Catalonia, respectively. These findings suggest that universal hepatitis A vaccination of children could reduce hepatitis A outbreak-associated costs.

## 1. Introduction

Hepatitis A is an acute inflammatory hepatic disease caused by the hepatitis A virus (HAV) [1]. HAV infections are associated with cases and outbreaks where asymptomatic, mild and severe hepatic diseases are reported [2]. In 2020, 170 million new cases were reported worldwide, causing 7134 deaths [3]. The epidemiology of hepatitis A is characterized by high infection rates in high-endemicity countries, where most cases occur in children, and they are often asymptomatic [4]. In Europe, hepatitis A endemicity is low, although it is higher in Northern and Eastern than in Southern and Western European countries [4]. In Spain, from 1995 to 2014, 5757 primary hospitalizations and 2273 secondary hospitalizations were registered, including 2114 patients with severe disease (hospitalized > 7 days), 168 patients with hepatic coma and 21 in-hospital deaths [5].

The risk of severe hepatitis A disease is higher in the adult population in countries with low endemicity, such as Spain, due to lower HAV circulation and a higher prevalence of susceptible adults [4,6]. In Spain, between 1995 and 2014, 76.7% of hepatitis A hospitalizations occurred among adults [5]. Therefore, implementing hepatitis A vaccination programs for children and screening and vaccination programs for adults can reduce the incidence of hepatitis A and the risk of severe disease among adults [7,8].

The World Health Organization (WHO) recommends the hepatitis A vaccine for susceptible individuals with a high risk of HAV infection and for post-exposure prophylaxis [9]. However, the WHO does not currently recommend universal hepatitis A vaccination or screening and vaccination programs for adults [8,9]. In contrast, the WHO suggests that universal hepatitis A vaccination for children aged over 1 year be implemented in countries with low endemicity based on the epidemiological situation and potential health benefits [9]. Despite these recommendations, only some countries and regions with low endemicity have implemented universal hepatitis A vaccination programs for pre-adolescents and children [10,11,12].

In Spain, hepatitis A vaccination is recommended for high-risk population groups, including patients with chronic liver disease, people traveling to countries with high rates of hepatitis A and people engaging in male-to-male sex or intravenous drug use [13]. However, since 1999, the region of Catalonia and the city of Ceuta have implemented hepatitis A vaccination programs targeting children and pre-adolescents [14]. Catalonia, an autonomous region with 8 million inhabitants, introduced the universal hepatitis A vaccination of pre-adolescents in 1999, and two doses of the hepatitis A vaccine have been administered to children aged 12–15 months and 6 years since 2014 [14]. Children aged 6 years vaccinated with only one dose of the vaccine receive the second dose at 11–12 years.

The aim of this study was to assess and compare hepatitis A outbreak-associated healthcare and epidemiological surveillance costs in Catalonia and a group of regions with a prevention strategy based on vaccinating only high-risk population groups during 2010–2018.

## 2. Materials and Methods

### 2.1. Study Design

The total, healthcare and epidemiological surveillance costs associated with hepatitis A outbreaks during 2010–2018 were assessed and compared in two types of regions implementing different hepatitis A vaccination strategies: (1) universal hepatitis A vaccination of children (children 12–15 months to 12 years) and vaccination of high-risk population groups and (2) vaccination of high-risk population groups. The following Spanish regions were included in the study: Catalonia, Castile and Leon, Murcia, Navarra, the Community of Madrid and the Community of Valencia. Catalonia implemented the first hepatitis A vaccination strategy [14]. Castile and Leon, Murcia, Navarra, the Community of Madrid and the Community of Valencia implemented the second hepatitis A vaccination strategy [14].

All hepatitis A outbreaks in Castile and Leon, Catalonia, the Community of Valencia, the Community of Madrid, Murcia and Navarre reported to the National Center of Epidemiology during 2010–2018 were included in the study. A hepatitis A outbreak was defined as two or more hepatitis A cases exposed to a common source of infection (epidemiological link) [14].

The comparison of hepatitis A outbreak-associated health costs in Catalonia and regions without universal hepatitis A vaccination of children was carried out in three phases. In the first phase, the hepatitis A outbreak-associated healthcare and epidemiological surveillance costs were determined from the number of hepatitis A outbreaks, hepatitis A outbreak-associated cases and hepatitis A outbreak-associated hospitalizations reported in the two types of regions during 2010–2018. In the second phase, the total, healthcare and epidemiological surveillance costs per million population were calculated in Catalonia and regions without universal hepatitis A vaccination of children. In the third phase, the ratios for total, healthcare and epidemiological surveillance costs per million population in regions without universal hepatitis A vaccination of children compared with Catalonia were calculated.

### 2.2. Healthcare Costs of Hepatitis A Outbreaks

The direct healthcare costs of hepatitis A outbreaks included hospitalization and primary healthcare costs. Healthcare costs due to hepatitis A outbreaks for each year from 2010 to 2018 were determined, taking into account the number of individuals hospitalized due to hepatitis A in each outbreak, the number of hepatitis A cases in each outbreak, the cost per hospital stay by age group due to hepatitis A in Spain, and public prices or tariffs per hospital day and per medical visit in Spain.

The hospitalization costs due to hepatitis A outbreaks during 2010–2018 were calculated for each year using the following data: (1) the number of hospitalized individuals by age group, (2) the median hospital stay for each age group in patients with hepatitis A in Spain from 2005 to 2008 [15] and (3) the tariff per hospital day in the normal ward in Spain from 2010 to 2018 [16]. The tariffs per hospital day for different years (2010 to 2018) were determined from the average tariff of EUR 727.66 per hospital day in the normal ward in Spain in 2019 [17] and the consumer price index variation for health services in Spain from 2010 to 2018 [16,18].

The hepatitis A outbreak-associated primary healthcare costs from 2010 to 2018 were calculated for each year using the following data: (1) the number of hepatitis A cases in different outbreaks and (2) the average public price per general practitioner visit with complementary tests from 2010 to 2018. It was assumed that all hepatitis A cases had received two medical visits with complementary tests. The prices per medical visit for different years from 2010 to 2018 were determined from the average public price of EUR 67.66 per medical visit with complementary tests in Spain in 2019 [17] and the consumer price index variation for health services in Spain from 2010 to 2018 [16,18].

### 2.3. Epidemiological Surveillance Costs of Hepatitis A Outbreaks

Epidemiological surveillance units are responsible for the control and monitoring of hepatitis A outbreaks. When a hepatitis A outbreak was reported, the following hepatitis A surveillance activities were performed: (1) epidemiological surveillance activities, (2) monitoring and outbreak control activities, (3) the collection and transport of biological samples from patients and potential sources of infectionto a reference laboratory, and (4) the microbiological analysis of biological samples. Epidemiological surveillance costs were determined for each year during the 2010–2018 period by using the following information: (1) personnel costs related to hepatitis A epidemiological surveillance activities in each hepatitis A outbreak, (2) costs for obtaining, transporting and processing biological samples and (3) hepatitis A detection costs.

The personnel costs related to hepatitis A epidemiological surveillance activities were determined using the following data: (1) the time necessary to undertake different epidemiological surveillance activities and (2) the cost per hour for technician work during 2010–2018 [16]. A questionnaire was developed to determine the time spent by technicians performing different epidemiological surveillance activities in participant epidemiological services. The costs per hour for technician work for different years (2010 to 2018) were determined from the monthly salaries of 22–23 civil service employees in Spain in 2011 (EUR 2499.3) [19] and the consumer price index variation for health services in Spain from 2010 to 2018 [16,18]. The cost per hour was determined assuming that the technicians worked 7.30 h per day and 22 days per month.

The hepatitis A outbreak detection costs were determined, taking into account that four samples were obtained, transported and analyzed in all outbreaks. The transport costs per sample in 2010–2018 were determined from a transport cost of EUR 65.7 in 2010 [19] and the consumer price index variation from 2010 to 2018 [17]. A price of EUR 32.75 was assumed for the hepatitis A polymerase chain reaction (PCR) test and EUR 45.5 for the combined hepatitis A immunoglobulin (Ig M) and PCR tests in 2010–2018 [20].

### 2.4. Total Costs of Hepatitis A Outbreaks

The total health costs of hepatitis A outbreaks in Catalonia and regions without universal hepatitis A vaccination of children were determined from the hepatitis A outbreak-associated healthcare and epidemiological surveillance costs in the two types of regions.

### 2.5. Statistical Analysis

The ratio of costs per million population in the group of regions without universal hepatitis A vaccination of children compared with Catalonia was calculated to compare the total, healthcare and epidemiological surveillance hepatitis A outbreak-associated costs in the two groups of regions. The total, healthcare and epidemiological surveillance costs per million population from 2010 to 2018 were determined in Catalonia and regions without universal hepatitis A vaccination of children by dividing their total, healthcare and epidemiological surveillance costs by their populations of 7,508,106 and 15,997,501 habitants (January 2015), respectively [21]. The populations of the participating regions accounted for 50% of the Spanish population. The ratio of costs per million population was calculated by dividing the costs per million population in regions without universal vaccination by the costs per million population in regions with this vaccination program.

The tendency over time from 2010 to 2018 was assessed in two ways: (1) Determining Pearson’s correlation coefficient between the variables “year” and “ratio of costs per million population” (total, healthcare, hospitalization, primary healthcare and epidemiological surveillance costs) in regions without universal hepatitis A vaccination of children versus Catalonia. A *p* < 0.05 was considered statistically significant. (2) Determining the ratios of costs per million population in regions without universal hepatitis A vaccination of children versus Catalonia in 2015–2018 and 2010–2014.

A sensitivity analysis was performed to assess the consistency of the results obtained in the study when the values assumed for the key variables varied by ±10% [16]. The following values were taken into account in the sensitivity analysis: EUR 800.43 and EUR 654.89 for the tariffs per hospital day; EUR 74.43 and EUR 60.89 for the public price per general practitioner visit with complementary tests; EUR 18.4 and EUR 15 for the personnel costs per hour (epidemiological surveillance activities); 1855 and 1512 for hepatitis A cases; and 483 and 395 for hospitalized hepatitis A cases.

The variables with the highest effect on the study results were those for which a 10% variation was associated with a greater variation in the ratio of hepatitis A outbreak-associated costs per million population (regions without universal hepatitis vaccination of children vs. Catalonia).

The statistical analysis was carried out using IBM-SPSS Version 18 (IBM-SPSS, Chicago, IL, USA) and Microsoft Excel Version 2016 (Microsoft Corporation, Redmond, WA, USA).

## 3. Results

### 3.1. Healthcare and Epidemiological Surveillance Costs of Hepatitis A Outbreaks

Throughout the study period, the participant regions reported 446 hepatitis A outbreaks, resulting in 1637 cases and 435 hospitalizations due to hepatitis A. In regions without hepatitis A vaccination of children, 330 (74%) hepatitis A outbreaks, 1105 (67.5%) hepatitis A outbreak-associated cases and 375 (86.2%) hepatitis A outbreak-associated hospitalizations were reported, compared with 116 (26%) hepatitis A outbreaks, 532 (32.5%) outbreak-associated cases and 60 (13.8%) hepatitis A outbreak-associated hospitalizations in Catalonia.

The hepatitis A outbreak-associated total healthcare and epidemiological surveillance costs in 2010–2018 were EUR 1.62 million in regions without universal hepatitis A vaccination of children and EUR 0.47 million in Catalonia (Supplementary Table S1). The hepatitis A outbreak-associated healthcare costs were EUR 1.20 million in regions without universal hepatitis A vaccination of children and EUR 0.31 million in Catalonia (Supplementary Table S1). The hepatitis A outbreak-associated epidemiological surveillance costs were EUR 0.42 million in regions without universal hepatitis A vaccination of children and EUR 0.15 million in Catalonia (Supplementary Table S1).

The hepatitis A outbreak-associated hospitalization costs were EUR 1.1 million in regions without universal hepatitis A vaccination of children and EUR 0.24 million in Catalonia (Supplementary Table S2).

### 3.2. Healthcare Costs per Million Population of Hepatitis A Outbreaks 

The hepatitis A outbreak-associated hospitalization costs per million population were 2.27 times greater (EUR 66,424 vs. EUR 29,288) in regions without universal hepatitis A vaccination of children than in Catalonia (Table 1). The hospitalization costs per million population ratios ranged from 0.89 in 2012 to 8.82 in 2017 (Table 1).

The hepatitis A outbreak-associated primary healthcare costs in regions without universal hepatitis A vaccination of children were 0.98 times (EUR 9076.1 vs. EUR 9228.4) those in Catalonia (Table 1). The primary healthcare costs per million population ratios ranged from 0.13 in 2016 to 4.71 in 2017 (Table 1).

The hepatitis A outbreak-associated healthcare costs per million population were 1.96 times greater (EUR 75,500.1 vs. EUR 38,516.4) in regions without universal hepatitis A vaccination of children than in Catalonia (Table 2). The healthcare costs per million population ratios ranged from 0.81 in 2012 to 8.15 in 2017 (Table 2).

### 3.3. Epidemiological Surveillance Costs per Million Population of Hepatitis A Outbreaks

The technicians dedicated specific amounts of time to various epidemiological surveillance activities for hepatitis A outbreaks. This included 7 h for registering and confirming outbreaks, 24 h for epidemiological investigations, 37.5 min for completing the epidemiological questionnaire for each case, 7 h for assessing or inspecting locations and infection sources and 9 h for developing and analyzing outbreak base data. Additionally, the total time needed for epidemiological surveillance was 47 h per hepatitis A outbreak, plus the time required to collect epidemiological data from hepatitis A cases (37.5 min per case).

The personnel costs of hepatitis A outbreak-associated epidemiological surveillance activities per million population were 1.34 times higher (EUR 17,099 vs. EUR 12,795) in regions without universal hepatitis A vaccination of children than in Catalonia (Table 3). The laboratory costs for hepatitis A outbreak-associated epidemiological surveillance activities per million population were 1.35 times higher (EUR 9071 vs. EUR 6720) in Catalonia (Table 3). The total epidemiological surveillance costs per million population were 1.34 times higher (EUR 26,171 vs. EUR 19,515) in Catalonia (Table 2).

### 3.4. Total Health Costs per Million Population of Hepatitis A Outbreaks 

The total healthcare and epidemiological surveillance costs of hepatitis A outbreaks per million population were 1.75 times greater (EUR 101,671 vs. EUR 58,032) in regions without universal hepatitis A vaccination of children than in Catalonia (Table 2). The ratio of total costs per million population in regions without universal hepatitis A vaccination of children versus Catalonia ranged from 0.74 in 2012 to 6.40 in 2017 (Table 2).

### 3.5. Tendencies for Study Outcome over Time during 2010–2018

This study found that the ratios of hepatitis A outbreak-associated costs per million population in regions without universal hepatitis A vaccination of children compared to Catalonia tended to increase over time from 2010 to 2018. First, moderate positive correlations were found between the variable “year” and different ratios of costs per million population: 0.56 for total costs, 0.46 for healthcare costs, 0.48 for hospitalization costs, 0.65 for primary healthcare costs and 0.79 for epidemiological surveillance costs. However, the correlation coefficient was statistically significant only for the epidemiological surveillance costs (*p* = 0.011).

Second, the ratios comparing costs per million population in regions without universal hepatitis A vaccination of children and Catalonia calculated for 2015–2018 were higher than those calculated for 2010–2014. In 2015–2018, the ratios were 2.68 times greater for total costs, 2.86 times greater for healthcare costs and 2.21 times greater for epidemiological surveillance costs in regions without universal hepatitis A vaccination of children than in Catalonia (Table 4). In 2015–2018, the ratios of hospitalization and primary healthcare costs per million population were 3.66 times greater (EUR 47,896 vs. EUR 13,083) and 1.03 times greater (EUR 5911 vs. EUR 5742) in regions without universal hepatitis A vaccination of children than in Catalonia. In 2010–2014, the ratio of healthcare costs per million population was 1.10 times greater in regions without universal hepatitis A vaccination of children than in Catalonia, although the ratios for total costs and epidemiological surveillance costs per million population were lower in regions without universal hepatitis A vaccination of children than in Catalonia (Table 4).

### 3.6. Sensitivity Analysis

The sensitivity analysis demonstrated a high level of consistency in the study results. The ratio for hepatitis A outbreak-associated total healthcare and epidemiological surveillance costs per million population (regions without universal hepatitis A vaccination of children vs. Catalonia) ranged from 1.70 to 1.78 when the values assumed for the study variables varied by ±10% (Table 5). This resulted in a variation of less than ±3% in the study outcome when the values of study variables varied by ±10%.

Among the variables included in the sensitivity analysis, “number of hospitalized hepatitis A cases” and “tariff per hospital day” had the highest impact on the study results. However, the ratios obtained in the sensitivity analysis for these variables varied by less than ±3% when their values varied by ±10% (Table 5). For the other variables included in the sensitivity analysis, the study outcome varied by less than ±2% when their values varied by ±10% (Table 5).

## 4. Discussion

This study highlights the potential economic benefits of implementing a universal hepatitis A vaccination strategy, in which all children are vaccinated, compared with a strategy based on vaccinating only high-risk populations. The findings revealed that the total, healthcare and epidemiological surveillance costs per million population associated with hepatitis A outbreaks were 75%, 96% and 34% higher, respectively, in the group of Spanish regions without a universal hepatitis A vaccination strategy than in Catalonia, where the hepatitis A prevention strategy is based on both universal hepatitis A vaccination of children and vaccination of high-risk population groups. In addition, the ratios of hepatitis A outbreak-associated costs per million population in regions without universal hepatitis A vaccination of children versus Catalonia tended to increase over time during 2010–2018. Thus, implementing a universal hepatitis A vaccination strategy could lead to a reduction in hepatitis A outbreak-associated healthcare and epidemiological surveillance costs.

This study found that implementing a hepatitis A prevention strategy including both hepatitis A vaccination of children and vaccination of high-risk groups could achieve a great reduction in hepatitis A outbreak-associated hospitalization costs, as they were 2.27 times greater in the group of Spanish regions without a universal hepatitis A vaccination strategy than in Catalonia. In addition, implementing this prevention strategy could reduce hepatitis A outbreak-associated epidemiological surveillance costs, due to its effect in reducing hepatitis A outbreaks and hepatitis A cases.

A previous study found that the cumulative incidence of hepatitis A outbreaks was 33% lower (relative risk = 0.77, 95% confidence interval: 0.62–0.95) in regions with universal vaccination than in those with high-risk vaccination only [14]. Additionally, the median number of cases per outbreak was lower in regions with universal vaccination than in those with high-risk hepatitis A vaccination (0 cases vs. 1 case per outbreak, *p* < 0.001) [14]. However, this prior study did not compare the economic impact of hepatitis A outbreaks between regions with and without universal vaccination. The results found in this study revealed that implementing a universal hepatitis A vaccination program for children can reduce not only the number of hepatitis A outbreak-associated cases but also the healthcare and epidemiological surveillance costs associated with hepatitis A outbreaks.

The positive correlations found in this study between the variable “year” and different ratios of costs per million population and the higher ratios for 2015–2018 than for 2010–2014 in regions without universal hepatitis A vaccination of children compared to Catalonia can be explained by three main factors: (1) an increasing prevalence of protected individuals among children, adolescents and young adults over time due to hepatitis A vaccination in Catalonia [22,23]; (2) the herd immunity effect derived from hepatitis A vaccination, preventing hepatitis A cases and hospitalizations [8]; and (3) an increasing prevalence of susceptible individuals among children, adolescents and young adults over time in regions without universal hepatitis A vaccination of children due to improved sanitation and lower circulation of VHA [23].

The results of this study are consistent with those of prior studies conducted in other countries that assessed the impact of universal hepatitis A vaccination on hepatitis A outbreak-associated healthcare costs, hospitalizations and medical visits [24,25]. For example, a study conducted in the US that evaluated the impact of universal hepatitis A vaccination introduced in 1996 found that the hepatitis A outbreak-associated healthcare costs, hospitalizations and medical visits decreased by 68.1%, from USD 29.1 million in 1996 to USD 9.3 million in 2004 [24]. This reduction was attributed to a 68.5% decrease in hepatitis A hospitalization rates and a 41.5% decrease in medical visit rates from 1996 to 2004 [24]. The study also reported reductions in hepatitis A outbreak-associated medical visit rates for all age groups, ranging from 32.9% among individuals aged 65 years or older to 48.8% among those aged 0–17 years [24].

Other studies conducted in different countries have reported that universal hepatitis A vaccination programs for children can reduce the burden of hepatitis A in the population [22,24,25,26,27,28,29,30,31,32,33,34,35,36]. These studies have found that such programs are associated with reductions in hepatitis A incidence [22,25,26,27,28,29,30,31,32,33,34,35], fulminant hepatitis A incidence [35], hepatitis A hospitalizations [21,22,36], hepatitis A-associated medical visits [24] and hepatitis A mortality [37]. Several studies have reported hepatitis A incidence reductions ranging from 76% to 98% after implementing universal hepatitis A vaccination programs [22,25,26,27,28,29,30,31,32,33,34,35]. Additionally, a study conducted in the US found that the age-adjusted hepatitis A mortality rate per million persons decreased by 45%, from 0.51 in 1990–1995 to 0.28 in 2000–2004, following the implementation of the universal hepatitis A vaccination program [38]. While reductions in hepatitis A-associated hospitalizations and medical visits due to universal hepatitis A vaccination programs are expected to lead to reduced hepatitis A outbreak-associated healthcare costs, most evaluative studies of hepatitis A vaccines have not specifically assessed their economic impact.

This study did not compare the cost-effectiveness of universal hepatitis A vaccination of children because it was focused on comparing hepatitis A outbreak-associated health and epidemiological surveillance costs in regions with and without this vaccination program. However, evaluative model-based studies found that universal hepatitis A vaccination programs for children were associated with incremental cost-effectiveness ratios (versus no vaccination) lower than USD 30,000–20,000 per quality-adjusted life-year (QALY) gained or life-year gained (LYG) in different countries [39,40,41,42,43,44]. Model-based evaluative studies obtained incremental cost-effectiveness ratios of <USD 0 (cost saving) to USD 4829 per QALY gained in Argentina [40], USD 4984 per LYG in Chile [41], USD 4933 per QALY gained in Indonesia [42], and USD 21,007 per disability-adjusted life-year (DALY) averted in South Africa [43]. A study evaluating the cost-effectiveness of universal hepatitis A vaccination programs for children aged 12-15 months in the United States found that this vaccination strategy was cost-effective compared with the high-risk vaccination strategy including only children living in states with high hepatitis A incidence [44]. Consequently, based on the results obtained in evaluative studies, universal hepatitis A vaccination programs of children should be recommended based on the effectiveness in reducing healthcare costs and the cost-effectiveness of the hepatitis A vaccine.

This study has several limitations. First, healthcare costs were determined by taking into account the median hospital stay for different age groups in hospitalized patients due to hepatitis A in Spain during the 2005–2008 period [15]. A median hospital stay per case greater than that found in the Spanish study would result in total and healthcare costs in regions without universal hepatitis A vaccination that were more than 3.5 and 3.9 times higher, respectively, than in regions with universal hepatitis A vaccination. However, obtaining more precise data regarding hospital stays among hospitalized patients with hepatitis A during the reported outbreaks was not possible. Second, healthcare and epidemiological surveillance costs, which were determined for 2010–2018, could be different after 2018. Nevertheless, the disruption of hepatitis A epidemiological surveillance activities during the COVID-19 pandemic period in Spain makes it challenging to conduct a consistent analysis of hepatitis A outbreak costs after 2018. Third, healthcare costs were determined using information about hepatitis A outbreaks and hepatitis A outbreak-associated cases and hospitalizations reported by Catalonia and the group of regions without universal hepatitis A vaccination of children included in the study. The ratios comparing the total healthcare and epidemiological surveillance costs in Catalonia and the group of regions without universal hepatitis A vaccination of children could be lower than those found in this study if a number of hepatitis A cases were not detected and reported in Catalonia. Nevertheless, similar underreporting rates could be assumed for Catalonia and regions without universal hepatitis A vaccination of children, and the sensitivity analysis demonstrated a high level of consistency in the study results.

## 5. Conclusions

The total, healthcare and epidemiological surveillance costs per million population associated with hepatitis A outbreaks were higher in the group of Spanish regions without universal hepatitis A vaccination programs of children than in Catalonia during 2010–2018. Universal hepatitis A vaccination programs of children could reduce the total, healthcare and epidemiological surveillance costs of hepatitis A outbreaks.

## Figures and Tables

**Table 1:** Hospitalization and primary healthcare costs (EUR) of hepatitis A outbreaks per million population in regions without universal hepatitis A vaccination (UHV) of children and Catalonia. Spain, 2010–2018.

	Hospitalization Costs (EUR) per Million Population			Primary Healthcare Costs (EUR) per Million Population		
Year	Regions without UHV of Children	Catalonia (with UHV of Children)	Ratio ^a^	Regions without UHV of Children	Catalonia (with UHV of Children)	Ratio ^a^
2010	3106.4	1241.0	2.50	699.3	502.1	1.39
2011	2903.8	2706.8	1.07	435.0	847.0	0.51
2012	2505.5	2802.7	0.89	427.1	827.3	0.52
2013	4463.2	3471.0	1.29	788.5	689.7	1.14
2014	5549.3	5983.5	0.93	814.8	619.9	1.31
2015	5557.2	5013.2	1.11	732.7	744.9	0.98
2016	4113.0	0.0	−	539.6	3998.4	0.13
2017	26,662.3	3022.8	8.82	2768.4	588.2	4.71
2018	11,563.4	5047.0	2.29	1870.7	411.1	4.55
Total	66,424.1	29,288.0	2.27	9076.1	9228.4	0.98

^a^ Ratio of costs (EUR) per million population between regions without universal hepatitis A vaccination of children and Catalonia (with universal hepatitis A vaccination of children).

**Table 2:** Total, healthcare and epidemiological surveillance costs (EUR) per million population of hepatitis A outbreaks per million population in regions without universal hepatitis A vaccination (UHV) of children and Catalonia. Spain, 2010–2018.

	Healthcare Costs (EUR) per Million Population			Epidemiological Surveillance Costs (EUR) per Million Population			Total Costs (EUR) per Million Population		
Year	Regions without UHV of Children	Catalonia (with UHV of Children)	Ratio ^a^	Regions without UHV of Children	Catalonia (with UHV of Children)	Ratio ^a^	Regions without UHV of Children	Catalonia (with UHV of Children)	Ratio ^a^
2010	3805.7	1743.1	2.18	2183.7	2466.9	0.89	5989.4	4210.0	1.42
2011	3338.8	3553.8	0.94	1701.0	2650.9	0.64	5039.8	6204.7	0.81
2012	2932.6	3630.0	0.81	1484.4	2344.2	0.63	4417.1	5974.2	0.74
2013	5251.7	4160.8	1.26	2772.7	2199.9	1.26	8024.4	6360.6	1.26
2014	6364.1	6603.3	0.96	1843.2	2521.0	0.73	8207.2	9124.3	0.90
2015	6289.9	5758.0	1.09	2378.8	2366.6	1.01	8668.7	8124.6	1.07
2016	4652.6	3998.4	1.16	1821.0	1136.6	1.60	6473.6	5135.0	1.26
2017	29,430.7	3610.9	8.15	7421.8	2145.6	3.46	36,852.5	5756.6	6.40
2018	13,434.1	5458.1	2.46	4564.4	1683.8	2.71	17,998.5	7141.9	2.52
Total Costs	75,500.1	38,516.4	1.96	26,171.1	19,515.5	1.34	101,671.2	58,031.9	1.75

^a^ Ratio of costs (EUR) per million population between regions without universal hepatitis A vaccination of children and Catalonia (with universal hepatitis A vaccination of children).

**Table 3:** Personnel and laboratory costs (EUR) of hepatitis A outbreak-associated epidemiological surveillance activities per million population in regions without universal hepatitis A vaccination (UHV) of children and Catalonia. Spain, 2010–2018.

	Personnel Costs (EUR) per Million Population			Laboratory Costs (EUR) per Million Population		
Year	Regions without UHV of Children	Catalonia (with UHV of Children)	Ratio ^a^	Regions without UHV of Children	Catalonia (with UHV of Children)	Ratio ^a^
2010	1449.8	1629.2	0.89	733.9	837.7	0.88
2011	1129.2	1764.8	0.64	571.8	886.1	0.65
2012	979.7	1551.7	0.63	504.8	792.5	0.64
2013	1802.5	1432.0	1.26	970.2	767.8	1.26
2014	1205.0	1634.3	0.74	638.1	886.7	0.72
2015	1545.7	1538.2	1.00	833.1	828.4	1.01
2016	1183.3	841.2	1.41	637.7	295.4	2.16
2017	4830.9	1314.6	3.67	2590.9	831.0	3.12
2018	2973.5	1089.1	2.73	1591.0	594.7	2.68
Total	17,099.6	12,795.2	1.34	9071.5	6720.3	1.35

^a^ Ratio of costs (EUR) per million population between regions without universal hepatitis A vaccination of children and Catalonia (with universal hepatitis A vaccination of children).

**Table 4:** Total, healthcare and epidemiological surveillance costs (EUR) per million population of hepatitis A outbreaks in regions without universal hepatitis A vaccination (UHV) of children and Catalonia in 2010–2014 and 2015–2018.

	Healthcare Costs (EUR) per Million Population			Epidemiological Surveillance Costs (EUR) per Million Population			Total Costs (EUR) per Million Population		
Periods	Regions without UHV of Children	Catalonia (with UHV of Children)	Ratio ^a^	Regions without UHV of Children	Catalonia (with UHV of Children)	Ratio ^a^	Regions without UHV of Children	Catalonia (with UHV of Children)	Ratio ^a^
2010–2014	21,692.8	19,691.0	1.10	9985.1	12,182.8	0.82	31,677.9	31,873.8	0.99
2015–2018	53,807.3	18,825.5	2.86	16,186.0	7332.6	2.21	69,993.3	26,158.1	2.68

^a^ Ratio of costs (EUR) per million population between regions without universal hepatitis A vaccination of children and Catalonia (with universal hepatitis A vaccination of children).

**Table 5:** Sensitivity analysis assessing the consistency of results obtained in the study for the ratio of total health costs per million population in regions without universal hepatitis A vaccination (UHV) of children versus Catalonia when the values of study variables varied by ±10%. Spain, 2010–2018.

Variable	Values 10% Greater and 10% Lower than the Study Values ^a^		Cost (EUR) per Million Population of Hepatitis A Outbreaks		Ratio of Costs (EUR) per Million Population in Regions without UHV of Children vs. Catalonia Ratio %	
			Regions without Universal Hepatitis A Vaccination of Children	Catalonia (with UHV of Children)		
Study results			101,671.2	58,031.9	1.75	−
Tariff per hospital day	+10%	800.43	108,251.6	60,946.5	1.78	1.7
	−10%	654.89	94,976.9	55,091.1	1.73	−1.7
Public price per general practitioner visit with complementary tests	+10%	74.43	102,537.6	58,859.1	1.74	0.6
	−10%	60.89	100,808.1	57,180.9	1.76	−0.6
Cost per hour for personnel costs	+10%	18.4	103,379.3	59,291.2	1.74	−0.6
	−10%	15.0	99,963.2	56,772.6	1.76	0.6
No. of hospitalized hepatitis A cases	+10%	478 ^b^	108,250.8	60,946.3	1.78	1.7
	−10%	391 ^c^	94,920.9	55,091.3	1.72	−1.7
No. of hepatitis A cases	+10%	1800 ^d^	102,688.6	59,647.8	1.74	−0.6
	−10%	1473 ^e^	100,800.2	57,165.1	1.76	0.6

^a^ Values assumed in the study (base analysis): EUR 727.66 for tariff per hospital day; EUR 67.66 for public price per general practitioner visit with complementary tests; EUR 16.7 per hour for personnel costs; 435 for hospitalized hepatitis A cases (375 in regions without hepatitis A vaccination of children and 60 in Catalonia); 1637 for hepatitis A cases (1105 in regions without hepatitis A vaccination of children and 532 in Catalonia). ^b^ 412 hospitalizations in regions without universal hepatitis A vaccination of children; 66 hospitalizations in Catalonia. ^c^ 337 hospitalizations in regions without universal hepatitis A vaccination of children; 54 hospitalizations in Catalonia. ^d^ 1215 cases in regions without universal hepatitis A vaccination of children; 585 cases in Catalonia. ^e^ 994 cases in regions without universal hepatitis A vaccination of children; 479 cases in Catalonia.

## Data Availability

The data presented in this study are available on request from the corresponding author.

## Supplementary Materials

The following are available online at https://www.mdpi.com/article/10.3390/vaccines12060648/s1: Table S1: Total, healthcare and epidemiological surveillance costs (EUR) of hepatitis A outbreaks (EUR) in regions without universal hepatitis A vaccination (UHV) of children and Catalonia. Spain, 2010–2018. Table S2: Hepatitis A outbreak-associated hospitalization and primary healthcare costs (EUR) in regions without universal hepatitis A vaccination (UHV) of children and Catalonia. Spain, 2010–2018.
