# Supplementary material for: Healthcare and Epidemiological Surveillance Costs of Hepatitis A Outbreaks in Spain in Regions with and without Universal Hepatitis A Vaccination of Children during 2010-2018
Source: Vaccines (Basel). 2024 Jun 11;12(6):648. doi: 10.3390/vaccines12060648 (PMC11209101; doi:10.3390/vaccines12060648)
Supplement: Supplementary file 1 [file vaccines-12-00648-s001.zip › vaccines-3032466-supplementary.pdf]

Supplementary Material

Supplementary Table S1. Total, healthcare and epidemiological surveillance costs (€) of hepatitis A outbreaks in regions without universal hepatitis A vaccination (UHV) of children and Catalonia. Spain, 2010–2018

	Healthcare costs (€)		Epidemiological surveillance costs (€)		Total costs (€)	
Year	Regions without UHV of children	Catalonia (with UHV of children)	Regions without UHV of children	Catalonia (with UHV of children)	Regions without UHV of children	Catalonia (with UHV of children)
2010	60881.1	13087.2	34933.9	18521.6	95815.0	31608.8
2011	53411.9	26682.2	27212.3	19903.4	80624.2	46585.6
2012	46914.5	27254.5	23747.4	17600.3	70661.9	44854.8
2013	84014.1	31239.4	44357.0	16516.8	128371.0	47756.2
2014	101809.3	49579.6	29485.9	18928.0	131295.2	68507.6
2015	100622.2	43233.8	38054.9	17768.7	138677.1	61002.5
2016	74430.4	30012.8	29131.0	8533.8	103561.4	38546.6
2017	470817.9	27111.3	118730.3	16109.5	589548.2	43220.9
2018	214912.2	40980.2	73019.3	12642.0	287931.5	53622.2
Total	1207813.5	289185.3	418672.1	146524.2	1626485.6	435709.5

Supplementary Table S2. Hepatitis A outbreak-associated hospitalisation and primary healthcare costs (€) in regions without universal hepatitis A vaccination (UHV) of children and Catalonia. Spain, 2010–2018

	Hospitalisation costs (€)		Primary Health care costs (€)	
Year	Regions without UHV of children	Catalonia (with UHV of children)	Regions without UHV of children	Catalonia (with UHV of children)
2010	49694.1	9317.6	11187.0	3769,5
2011	46453.0	20323.2	6958.9	6359,0
2012	40081.8	21042.9	6832.7	6211,5
2013	71400.0	26061.0	12614.1	5178,4
2014	88775.3	44924.6	13034.0	4653,9
2015	88900.6	37639.4	11721.6	5592,6
2016	65798.4	0.0	8632.0	30020,0
2017	426530.1	22695.4	44287.8	4415,9
2018	184985.6	37893.5	29926.6	3086,8
Total	1062618.8	219897.6	145194.7	69287,7
